# The Complex Interplay between DNA Injury and Repair in Enzymatically Induced Mutagenesis and DNA Damage in B Lymphocytes

**DOI:** 10.3390/ijms18091876

**Published:** 2017-08-30

**Authors:** Mahnoush Bahjat, Jeroen E. J. Guikema

**Affiliations:** Department of Pathology, Academic Medical Center, University of Amsterdam; Lymphoma and Myeloma Center Amsterdam (LYMMCARE), Amsterdam 1105 AZ, The Netherlands; m.bahjat@amc.uva.nl

**Keywords:** B lymphocyte, V(D)J recombination, immunoglobulin (Ig), class switch recombination (CSR), somatic hypermutation (SHM), recombination activating gene products 1 & 2 (RAG1, RAG2), activation-induced cytidine deaminase (AID), DNA repair, DNA damage response (DDR)

## Abstract

Lymphocytes are endowed with unique and specialized enzymatic mutagenic properties that allow them to diversify their antigen receptors, which are crucial sensors for pathogens and mediators of adaptive immunity. During lymphocyte development, the antigen receptors expressed by B and T lymphocytes are assembled in an antigen-independent fashion by ordered variable gene segment recombinations (V(D)J recombination), which is a highly ordered and regulated process that requires the recombination activating gene products 1 & 2 (RAG1, RAG2). Upon activation by antigen, B lymphocytes undergo additional diversifications of their immunoglobulin B-cell receptors. Enzymatically induced somatic hypermutation (SHM) and immunoglobulin class switch recombination (CSR) improves the affinity for antigen and shape the effector function of the humoral immune response, respectively. The activation-induced cytidine deaminase (AID) enzyme is crucial for both SHM and CSR. These processes have evolved to both utilize as well as evade different DNA repair and DNA damage response pathways. The delicate balance between enzymatic mutagenesis and DNA repair is crucial for effective immune responses and the maintenance of genomic integrity. Not surprisingly, disturbances in this balance are at the basis of lymphoid malignancies by provoking the formation of oncogenic mutations and chromosomal aberrations. In this review, we discuss recent mechanistic insight into the regulation of RAG1/2 and AID expression and activity in lymphocytes and the complex interplay between these mutagenic enzymes and DNA repair and DNA damage response pathways, focusing on the base excision repair and mismatch repair pathways. We discuss how disturbances of this interplay induce genomic instability and contribute to oncogenesis.

## 1. B-Cell Development

**Figure 1:**
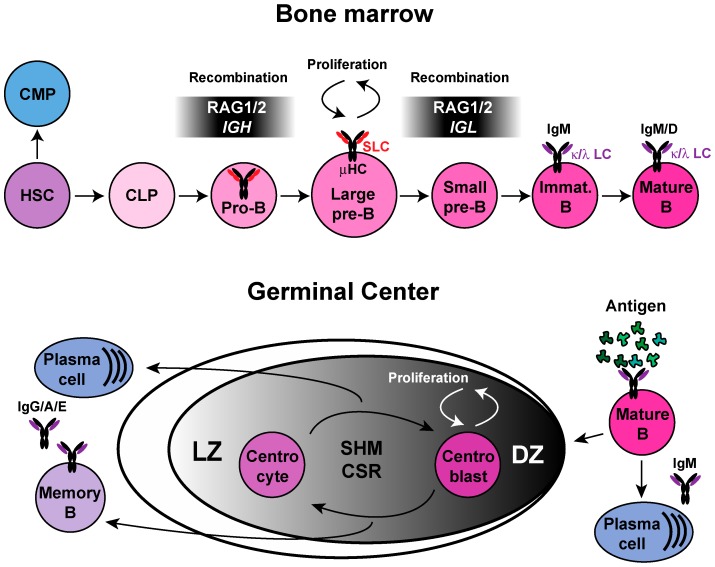
Antigen-independent B-cell development in the bone marrow, and antigen-dependent B-cell activation and differentiation in the germinal center. B cells develop in an antigen-independent fashion from common lymphocyte progenitors (CLP) in the bone marrow. The recombination activating gene 1/2 complex (RAG1/2) is expressed in pro-B cells, which, when leading to successful immunoglobulin (*Ig*) heavy chain recombination and expression, results in the formation of the pre-B cell receptor (pre-BCR) upon pairing with the surrogate light chain (SLC). Clonal expansion takes place in large pre-B cells, where RAG1/2 expression is downregulated. Pre-BCR signaling is involved in cell-cycle exit, re-expression of RAG1/2, and *Ig* light chain recombination. Upon expression of a complete IgM molecule, B cells fully mature and exit the bone marrow. Antigen-dependent B-cell activation in secondary lymphoid organs initiates the germinal center reaction, where antigen-specific B cells undergo affinity maturation through iterative rounds of *Ig* gene somatic hypermutations (SHM), and undergo *Ig* class switch recombination (CSR). Antigen-dependent selection leads to memory B-cell and plasma cell differentiation.

B-cell precursors are generated from hematopoietic stem cells (HSCs) in the liver during the fetal life and postnatally in the bone marrow (BM) through an ordered developmental pathway [1]. The differentiation pathway from HSCs to mature B cells can be divided into several stages. HSCs give rise to two predominant populations: the common myeloid progenitor (CMP) and the common lymphocyte progenitor (CLP). It is from CLPs that B cells arise [2] (Figure 1). The expression of B lineage-associated genes, as well as the survival and expansion of committed B-cell progenitors, is controlled by transcriptional factors including PU.1, Ikaros, E2A, EBF, and Pax5 [3,4]. In the BM, committed B cells develop from pro-B-cells to pre-B cells and from pre-B cells to immature B cells [5]. The early phase of B-cell differentiation is characterized by the assembly of the immunoglobulin (*Ig*) heavy-chain (*H*) and light-chain (*L*) gene segments through the process of V(D)J recombination, which brings together three gene segments termed: variable (V), diversity (D), and joining (J) segments [6,7].

At the pro-B-cell stage, the recombination activating genes 1 and 2 complex (RAG1/2) initiates *DJ* joining in the *IgH* locus, and is required for B-cell development [8]. Joining of a *V* segment to the *DJ* leads to the expression of membrane μ chains (μH) with surrogate light chains (SLC). As soon as μH chain proteins appear in the cytoplasm and can be assembled into a functional precursor B-cell receptor (pre-BCR), pre-B cells develop into large pre-B cells where RAG1/2 expression is downregulated and cells undergo several rounds of division. Following proliferation, RAG expression is upregulated again in small pre-B cells resulting in κ light chain (*Igκ*) recombination [9,10] (Figure 1). The expression of a tolerant (non-self) functional antigen receptor switches off RAG expression. However, in lymphocytes with autoreactive receptors the rearrangement continues by prolonged expression of RAG, which allows the secondary light chain recombination in a process known as receptor editing [11].

In the *Ig* loci, both *H* chain alleles begin to rearrange and successful *V* to *DJ* rearrangement on one allele suppresses further rearrangement on the other allele. *Igκ* chain rearrangement proceeds in a similar manner, and only if rearrangement of both alleles of the *Igκ* locus has been non-productive, then rearrangement takes place on the *Igλ* locus. This process is called allelic exclusion [12].

Immature B cells subsequently express a complete IgM molecule on their surface and undergo selection for self-tolerance. B cells leave the BM at the transitional B-cell stage and complete their final development into mature B cells in the periphery [7]. Upon encountering antigen in secondary lymphoid organs, B cells become activated and differentiate into memory B cells or antibody-secreting plasma cells. These processes occur in the germinal center (GC), a specialized anatomical site that arises within lymphoid follicles, where Ig receptors undergo somatic hypermutation (SHM) and class switch recombination (CSR). The germinal center contains two zones: a dark zone (DZ) and a light zone (LZ). In the DZ, large centroblasts that are rapidly proliferating undergo somatic hypermutation (SHM) and clonal expansion [13] (Figure 1). During SHM, single nucleotide substitutions are introduced at the rearranged *V(D)J* gene segment at rates of 10^−3^ to 10^−5^ mutations per base pair per generation [14]. Later on in the LZ, centrocytes undergo selection based on their competitiveness for antigen binding on follicular dendritic cells (FDCs) and depending on the signal that they receive from T follicular helper cells (Tfh), they may survive, differentiate, or undergo apoptosis [15]. In addition to SHM, B cells can undergo Ig class switch recombination (CSR), the process by which B cells rearrange regions of the *IgH* locus to switch from expressing one upstream class of immunoglobulin (such as IgM) to a downstream one (such as IgG). Thus, several days after the first encounter with antigen, low-affinity IgM antibodies specific for the antigen and high-affinity switched antibodies, typically of an IgG, IgA, or IgE isotype, are produced and secreted [16].

By these mechanisms, the generation of a vast repertoire of antibodies is ensured. However, each of these processes (V(D)J recombination, CSR and SHM) is characterized by the programmed induction of different forms of DNA damage, catalyzed by specialized enzymes. Highly efficient DNA repair systems would in principle thus counteract *Ig* diversification mechanisms. However, in B cells, several mechanisms have evolved that evade faithful DNA repair, or alter the fidelity of DNA repair. In this review, we focus on the involvement and effects of DNA repair pathways during these three genetic alteration events, and discuss the most recent findings on how DNA repair mechanisms shape the humoral immune repertoire and may be involved in B-cell lymphomagenesis.

## 2. RAG and V(D)J Recombination

**Figure 2:**
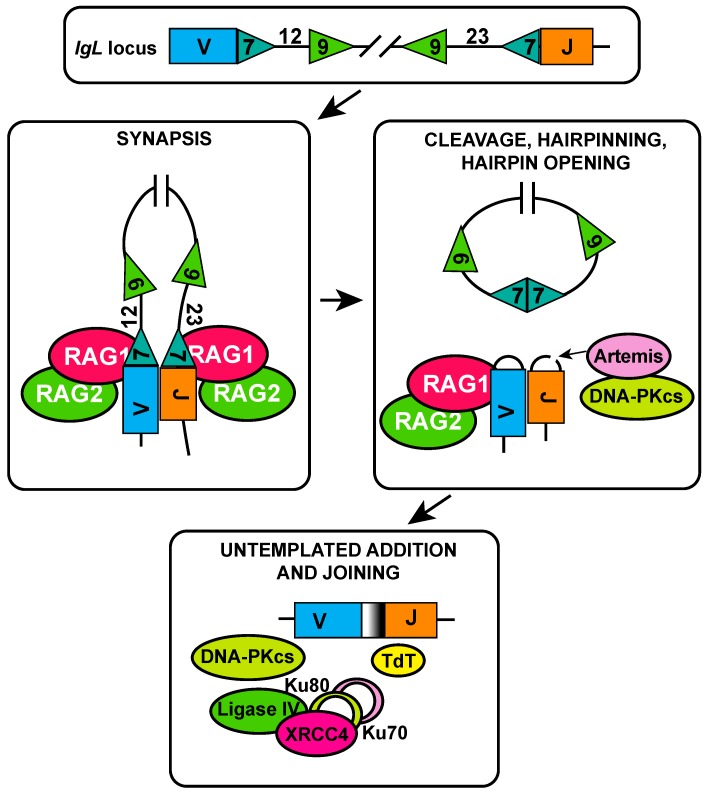
Molecular mechanism of V(D)J recombination. Schematic representation of immunoglobulin light chain loci (*IgL*) showing a *V* gene segment flanked by 12-base pair (bp) spacer recombination signal sequence (RSS) and a *J* gene segments flanked by a 23-bp spacer RSS. The recombination activating gene 1/2 complex (RAG1/2) forms a synaptic complex with a 12-bp spacer RSS and a 23-bp spacer RSS, resulting in nicking and hairpin formation at the coding ends. RSS heptamers are joined to form a signal joint. Artemis is required for coding end hairpin opening and is activated by the DNA protein kinase catalytic subunit (DNA-PKcs). Upon hairpin opening, terminal deoxynucleotidyl transferase (TdT) adds untemplated nucleotides and increases junctional diversity. Ends are sealed by non-homologous end joining (NHEJ), requiring Ku70 (depicted in pink), Ku80 (depicted in light green), XRCC4, DNA ligase IV, and DNA-PKcs.

V(D)J recombination can be divided into two phases, a cleavage phase and a joining phase. The cleavage phase requires RAG1/2 endonuclease activity, which creates double-strand breaks (DSBs) at recombination signal sequences (RSS) adjacent to each antigen receptor gene segment. RSS are highly conserved and are composed of a heptamer and a nonamer sequence separated by a less conserved spacer of either 12 or 23 nucleotides (nts). Upon binding to an RSS, RAG first introduces a nick between the coding flank and the heptamer, the liberated 3′-hydroxyl group performs a nucleophilic attack on the other strand, producing a hairpin structure at the coding end by transesterification, and a blunt DSB at the signal end (RSS flank) [17]. Recombination can only take place between gene segments that are flanked by an RSS with spacers of different length (12/23 rule), to avoid inappropriate recombinations (e.g., *V* to *V*). The 12/23 rule is enforced by the formation of a synaptic complex, as hairpinning only takes place in case both a 12-spacer and a 23-spacer RSS are bound [17,18]. Recombination is preceded by hairpin opening, which requires the Artemis endonuclease. Functional Artemis deficiency in humans results in the absence of B and T cells and hypersensitivity to DNA damage, underscoring its crucial importance for V(D)J recombination [19]. The hairpin opening capacity of Artemis is activated by DNA protein kinase catalytic subunit (DNA-PKcs) phosphorylation, which orchestrates the non-homologous end joining (NHEJ) functions required for V(D)J recombination [19,20] (Figure 2).

During assembly of the *Ig* gene segments, the repertoire is further diversified by untemplated addition of nts at the junctions of the gene segments. Terminal deoxynucleotidyl transferase (TdT) is the DNA polymerase responsible for the junctional addition of nts to single-stranded DNA during V(D)J recombination, allowing a near limitless array of antigen receptors of unique specificity to be generated [19,21]. Expressed components of the NHEJ DNA repair pathway then carry out the joining reaction [8,22,23], relying on the cofactors Ku70, Ku80, XRCC4, and DNA ligase IV, which are all required for V(D)J recombination [24,25] (Figure 2). The recently identified XRCC4 cofactors XRCC4-like factor (XLF) and paralog of XRCC4 and XLF (PAXX) were shown to be partially redundant as only combined deficiency of these factors resulted in impaired joining of RAG cleaved DNA ends [26]. During V(D)J recombination, the cleavage and joining phases take place in a highly coordinated and concerted fashion, ensuring the proper resolution of RAG-initiated DSBs and preventing illegitimate recombinations that may result in chromosomal translocations. Not surprisingly, next to a tight regulation of DSB repair during V(D)J recombination, the expression and activity of RAG itself is also subject to intense regulation.

## 3. Regulation of RAG1 and RAG2 Expression and Activity

There are several mechanisms to ensure proper RAG regulation during lymphocyte development. An important mechanism to keep RAG-mediated DSBs in check is via transcriptional regulation of the *RAG1/2* genes. The RAG complex is a lymphoid-cell-specific enzyme and is expressed in a narrow developmental window in B and T lymphocytes [27]. Using a luciferase reporter construct, Lauring and Schlissel showed RAG activity in various B- and T-cell lines, but little activity in nonlymphoid cells [28]. *RAG1* and *RAG2* are transcribed from the same locus. Sequencing of the genomic DNA surrounding the *RAG* genes, in combination with in vitro and in vivo experiments, led to the identification of *cis*-elements including the proximal, distal enhancer and the *RAG* promoters that are required for *RAG* regulation in lymphocytes [29]. The binding sites of various transcription factors including paired box 5 (PAX5), MYB, SP1, lymphoid enhancer binding factor 1 (LEF1), NF-Y, C/EBP, and GATA3 to the *RAG* promoter have been identified and confirmed by in vivo footprinting assays and chromatin immunoprecipitations [28,30,31]. The *RAG* enhancer (*Erag*) is highly conserved and was shown to be essential for RAG expression [32]. Various transcription factors were shown to bind to *Erag*, including forkhead box O1 (FOXO1), forkhead box P1 (FOXP1), PAX5, nuclear factor kappa B (NF-κB), and Ikaros, and regulate *RAG* transcription [33]. Although RAG1/2 seem to be coordinately regulated at the transcriptional level, post-transcriptional regulation differs, as, unlike RAG1, RAG2 is subject to cell-cycle-dependent periodic degradation. Phosphorylation of threonine 490 of RAG2 by the CyclinA–cyclin dependent kinase 2 (CDK2) complex, which is active at the G1/S transition of the cell cycle causes its ubiquitination and subsequent degradation by Skp2-SCF E3 ubiquitin ligase complex and in this way restrict its expression to the G1 phase [34,35].

Another mechanism that is involved in the coordination and cell type specificity of V(D)J recombination is the accessibility of chromatin at target gene segments [36]. At the *Ig* loci, the accessibility of the chromatin is governed by the concerted action of enhancers and promoters. These accessibility control elements (ACEs) are required for the recruitment of enzymes that covalently modify or remodel nucleosomes and therefore make the chromatin of target genes accessible [36]. The role of chromatin structure was confirmed by using recombinant RAG proteins on naked DNA or chromatin. Whereas RAG cleavage of RSS-containing gene segments took place on naked DNA, RSS cleavage on chromatin depended on the cell type of origin [37]. DNA methylation, histone modifications, and nuclear localization are three potential mechanisms that are required for chromatin accessibility during V(D)J recombination [38]. RAG2 contains a plant homeodomain (PHD) finger that is required for the recognition of tri-methylated lysine 4 on histone H3 (H3K4me3) [39]. Abrogation of this domain severely impaired V(D)J recombination [40]. This topic has been reviewed extensively elsewhere [38]. Together, all these mechanisms function to restrict RAG activity to the right time and place.

## 4. Signaling Pathways Involved in RAG1 and RAG2 Expression

**Figure 3:**
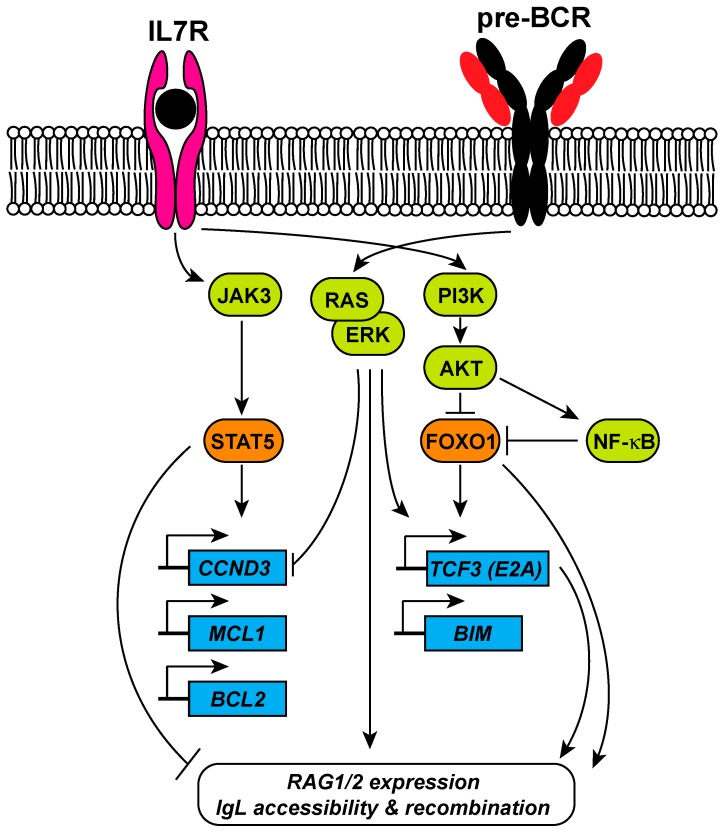
Signaling events downstream of IL7R (depicted in purple) and the pre-BCR (depicted in black and red) involved in the regulation of RAG1/2 expression and *Ig* light chain recombination. Coordinate signaling through the IL7 receptor (IL7R) and the pre B-cell receptor (pre-BCR) regulate the expression of the recombination activating gene 1/2 complex (RAG1/2) and immunoglobulin light chain (*IgL*) recombination in pre-B cells. The IL7R and the pre-BCR have opposing functions in pre-B cells. Engagement of the IL7R results in activation of signal transducer and activator of transcription 5 (STAT5) that promotes proliferation and survival by driving the expression of cyclin D3 (*CCND3*), and the anti-apoptotic genes *BCL2* and *MCL1*. Simultaneously, IL7R signaling activates the phosphoinositide 3-kinase (PI3K) and AKT, which negatively regulates the forkhead box O1 (FOXO1) transcription factor that is required for *RAG1/2* expression and the expression of the apoptotic *BIM* gene. In addition, *IgL* accessibility is inhibited by STAT5. Signaling through the pre-BCR activates the RAS and extracellular signal-regulated kinase (ERK) pathway, which induces *TCF3* expression (E2A) and represses *CCND3*. E2A binds and increases the accessibility of the *IgLκ* locus. Arrows represent positive regulation (activation); T-bars represent negative regulation (inhibition).

RAG expression has a dynamic pattern throughout B-cell development, in which phases of proliferation are separated from phases of recombination, in order to ensure genomic integrity [41]. Balance between the signaling pathways emanating from the pre-BCR and the interleukin-7 receptor (IL7R) are required for this dynamic pattern of RAG expression and activity [42,43]. In the BM, the developing B cells proliferate in response to IL7R signaling pathway through activation of janus kinase 3 (JAK3) and signal transducer and activator of transcription 5 (STAT5), which in turn stimulates the transcription of *Ccnd3* (encoding cyclin D3). Binding of cyclin D3 to Cdk4 and Cdk6 induces transition from the G1/S checkpoint of the cell-cycle and DNA replication [42]. In addition to stimulating proliferation, STAT5 enhances pro-B-cell survival by activating expression of anti-apoptotic genes encoding Mcl1 and Bcl2 [44]. STAT5 also has an inhibitory effect on *Igκ* recombination. Furthermore, activation of the PI3K-AKT signaling pathway by IL7 leads to phosphorylation and subsequent degradation of FOXO1, a major transcription factor for *RAG1/2* expression, and in this way inhibits recombination. Repression of FOXO1 also mediates IL7-induced pro-B-cell survival by inhibition of BIM expression [45]. In contrast, signaling from the pre-BCR represses proliferation and induces *IgL* recombination. Activation of RAS/ERK downstream of pre-BCR represses cyclin D3 and drives expression of the E2A transcription factor. E2A cooperates with interferon regulatory factor 4 (IRF4), downstream of B-cell linker protein (BLNK), to increase *Igκ* locus accessibility (Figure 3). The exact regulatory mechanism of these transcription factors downstream of IL7R and pre-BCR, and their involvement in early B-cell development, has been reviewed extensively and will not be covered here [42,46]. Altogether, the interplay between these two signaling pathways ensures the inhibition of RAG expression during proliferation in order to conserve genomic stability in pre-B cells.

Another signaling pathway that was implicated in the regulation of B-cell development and RAG expression is the NF-κB pathway. The exact functions of NF-κB in B-cell progenitors are still not completely clear, as several studies that addressed the role of NF-κB in early B-cell development presented conflicting results. It was shown by Scherer et al. that inhibition of NF-κB by using a trans-dominant form of I kappa B alpha (IκBα) led to inhibition of germline *Igκ* transcription and rearrangement but not of RAG activity [47]. On the other hand, NF-κB was shown to be dispensable for B-cell development, but promoted the survival of Igλ expressing B cells in the mouse by regulating expression of the anti-apoptotic Bcl2 protein [48,49,50]. To demonstrate the role of NF-κB in receptor editing, Cadera et al. created an IκBα + /lacZ reporter mice, in which an *IKBA* gene was replaced with an lacZ (β-gal) reporter. The study showed that β-gal + sorted pre-B cells have higher levels of various markers of receptor editing [51]. More recently, it was shown that NF-κB is active in pre-B cells and is required for receptor editing [52,53]. Strikingly, it was shown that NF-κB1/p50-deficient B cells express a higher level of RAG [52]. In addition, we recently reported a novel pathway that suppresses RAG expression in cycling-transformed human and mouse pre-B cells, which involves the negative regulation of FOXO1 by NF-κB [54]. It is known that in proliferating pre-B cells, RAG expression is repressed by AKT signaling that negatively regulates FOXO1. Our data suggested that the AKT signaling inhibits FOXO1 directly, and by feeding into NF-κB pathway, since simultaneous inhibition of the AKT and the NF-κB pathways resulted in increased levels of nuclear FOXO1 and a synergistic increase in Rag activity (Figure 3). Recently, Cdk4 was shown to have an inhibitory effect on *Rag* expression in B-cell lymphomas in *Eμ-Myc*-transgenic mice. The authors showed that Cdk4 deficiency accelerated lymphomagenesis in a Rag-dependent manner. Their data also showed that Cdk4 phosphorylated FOXO1 on serine 329 that resulted in its nuclear exclusion and degradation. [55]. Furthermore, IκBα was shown to bind to CDK4 and inhibits its kinase activity [56]. We showed that inhibition of NF-κB and AKT diminished *CDK4* mRNA and CDK4 protein levels, and therefore decreased FOXO1-serine 329 phosphorylation. We hypothesized that IκBα stabilization could promote *RAG* expression by inhibition of CDK4, which acts as a negative regulator of FOXO1 [54,55].

## 5. The DNA Damage Response Regulates RAG Activity

As the genomic integrity of cells is constantly threatened by DNA damage caused by extrinsic and intrinsic factors, several systems have evolved that detect and signal DNA damage to initiate and direct DNA repair [57,58]. This DNA damage response (DDR) is initiated by phosphoinositide 3-kinase-related protein kinases (PIKKs), including ataxia telangiectasia mutated (ATM), ataxia telangiectasia and rad3-related (ATR), and DNA-PKcs. These kinases directly sense the damage and activate other mediators that control cell-cycle progression, DNA repair, and apoptosis [58,59]. The DDR is intimately involved in all forms of programmed DNA damage during *Ig* gene alterations in lymphocytes, and regulates the fidelity and resolution of these alterations. Several mechanisms have evolved that, on the one hand, allow DNA damage necessary for these diversification mechanisms to occur and, on the other hand, keep overt DNA damage and recombination in check. As such, there exists an intricate interplay between the induction and repair of programmed DNA damage in lymphocytes, which, when dysregulated, may lead to genomic aberrations and malignancies. Cleavage by RAG, which normally occurs in the G1 phase, activates the DDR. Cleavage by RAG leads to the formation of four broken DNA ends, which are processed and joined by NHEJ. Cooperation of RAG and ATM is essential for holding these chromosomal ends together to facilitate their correct repair [60]. On the other hand, activation of p53 through ATM triggers the G1/S checkpoint and therefore suppresses the entrance of cells with unrepaired DSBs to the S-phase [61]. Moreover, upon RAG cleavage of *Igκ* loci, ATM negatively regulates RAG expression and in this way suppresses further *Igκ* rearrangements and enforces allelic exclusion [62]. It has been recently shown that ATM is involved in allelic exclusion of *IgH* recombination. ATM-deficient mice showed an increase in frequency of biallelic expression and aberrant *V*-to-*DJ* rearrangements [63,64].

Lack of ATM in both human and mice is involved in lymphoid malignancies that harbor chromosomal translocations involving antigen receptor genes [65,66,67,68]. It has been confirmed that development of these lymphoid malignancies is the result of RAG-induced DSBs, since ATM-deficient mice did not develop tumors in the absence of Rag [69].

**Figure 4:**
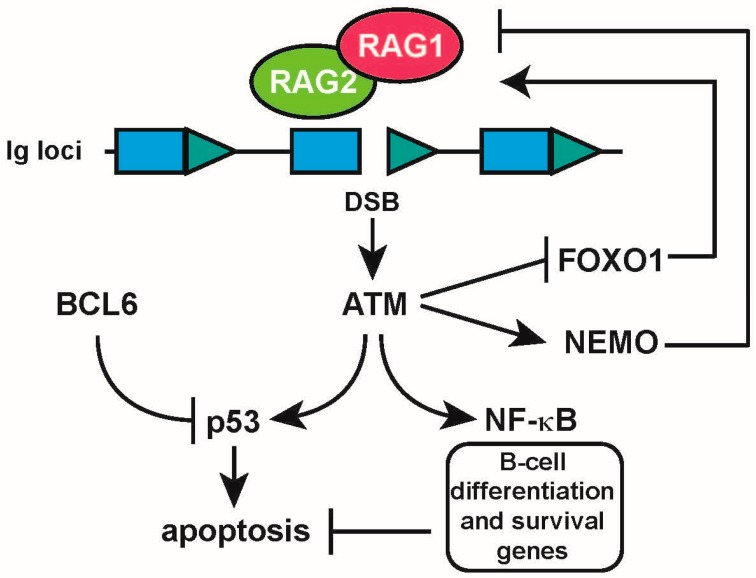
DNA damage regulates the expression of RAG1/2 through activation of the DDR. Cleavage of recombination signal sequences (RSS) by the recombination activating gene 1/2 complex (RAG1/2) (shown in green and red) in the *Ig* loci activates the ataxia telangiectasia mutated (ATM) kinase, which triggers a negative feedback loop involving the dissociation of forkhead box O1 (FOXO1) from the *Erag* enhancer in the *RAG1/2* locus, and the induction of the NF-κB essential modulator (NEMO), shutting off *RAG1/2* transcription. In parallel, a p53-dependent apoptotic response is triggered, which is counteracted by the B-cell lymphoma 6 (BCL6) protein to allow recombination to take place. At the same time, an NF-κB-dependent gene expression program that includes several key B-cell differentiation and survival genes is activated by ATM (indicated in the black frame). Arrows represent positive regulation (activation); T-bars represent negative regulation (inhibition).

Recently, we showed that exogenous DNA damage, such as treatment with the DNA damaging agents neocarzinostatin (NCS) or ionizing radiation (IR), repressed *RAG1/2* mRNA and RAG1 protein expression in an ATM-dependent manner in both human and mouse pre-B cells. We showed that induction of DNA damage resulted in the loss of FOXO1 binding to the *RAG1/2* enhancer region (*Erag*) and its subsequent phosphorylation and cleavage in an ATM-dependent manner in human pre-B-cell lines. However, ATM was shown not to be directly involved in FOXO1 phosphorylation. Rather, our results indicate that ATM regulated the dissociation of FOXO1 from the *Erag* enhancer, which consequently led to the downmodulation of *RAG1/2* mRNA, protein level, and activity [70]. In line with our findings, another group subsequently showed that treatment with IR and the DNA damaging agent etoposide resulted in an ATM-dependent repression of *Rag1* and *Rag2* expression in a similar fashion in primary mouse pre-B cells and pro-B cells. In addition, the work from the Bassing group also suggested the involvement of the NF-κB essential modulator (Nemo), as the loss of *Rag1/2* expression was partially rescued in Nemo-deficient B cells [71] (Figure 4). The exact mechanism by which Nemo regulates *Rag1/2* expression, however, remains to be established. We speculate that the rapid loss of *RAG1/2* expression in pre-B cells sustaining (exogenous) DNA damage is part of a protective mechanism that diminishes the risk of chromosomal translocations involving RAG-induced DSBs and DSBs caused by extrinsic factors (Figure 4).

Using NHEJ deficient (*Artemis−/−*, *Ku70−/−*, and *Scid*) Abelson kinase (Abl) transformed pre-B cells, the Sleckman group found that Rag-induced DSBs regulates the expression of a large number of genes, and activates a broad genetic program in developing pre-B cells, partially through the ATM-dependent activation of NF-κB, which in turn regulated the expression of several important B-cell differentiation factors [72]. These results argue that, in addition to being required for V(D)J recombination, RAG-dependent programmed DSBs can also be regarded as important developmental cues for B-cell development, implicating the DDR not only as a mechanism to detect and instigate repair of DNA damage but also as an important regulator of B-cell differentiation (Figure 4).

During B-cell development, the DDR should be properly regulated in order to protect pre-B cells from DNA damage-induced apoptosis during *Ig* gene rearrangements. Upon productive V-to-DJ rearrangement, signaling through pre-BCR induces *Bcl6* expression, which protects pre-B cells from DNA damage-induced apoptosis by negatively regulating genes that are involved in checkpoint activation including Arf, p53, p21, and p27 [73]. During *IgL* recombination, the DDR is activated, resulting in the upregulation of Arf and p53, which is counteracted by Bcl6. Consequently, in the absence of Bcl6, few small resting pre-B cells survive the transition from large cycling pre-B cell to small resting pre-B cell, leading to a diminished B-cell receptor repertoire, indicating that Bcl6 plays a crucial role in the generation of a diverse primary B-cell repertoire [73] (Figure 4).

## 6. Malignancies Associated with Dysregulated RAG Activity

A large fraction of recurrent chromosomal translocations found in B-cell lymphomas and leukemias involves the *Ig* loci and aberrant RAG activity, which underscores the importance of proper regulation of this potentially harmful enzyme [74,75,76,77,78]. Typical examples of these translocations are as follows: the t (11;14) *BCL1*/*IGH* translocation in mantle zone lymphoma (MCL) and the t (14;18) *BCL2*/*IGH* translocation in follicular lymphoma (FL) [74]. Rag-induced DSBs can also cause *IgH/c-Myc* translocations in leukemic pro-B cells in p53-deficient mice [79]. Aberrant RAG activity can be a genomic threat especially to B cells that are actively engaged in V(D)J recombination. A recent study by Papaemmanuil et al. suggested that aberrant RAG activity is a dominant driver of secondary genetic hits in *ETV6-RUNX1* + childhood acute lymphoblastic leukemia (ALL), where many breakpoints map near RSS motifs [80]. In addition, ongoing rearrangement events at the *IGH* locus have been observed in ALL after malignant transformation, as a result of constitutive *RAG* expression, which may contribute to the evolution of secondary genetic hits [81].

## 7. The Mechanism of Class Switch Recombination and Involvement of Base Excision Repair (BER)

**Figure 5:**
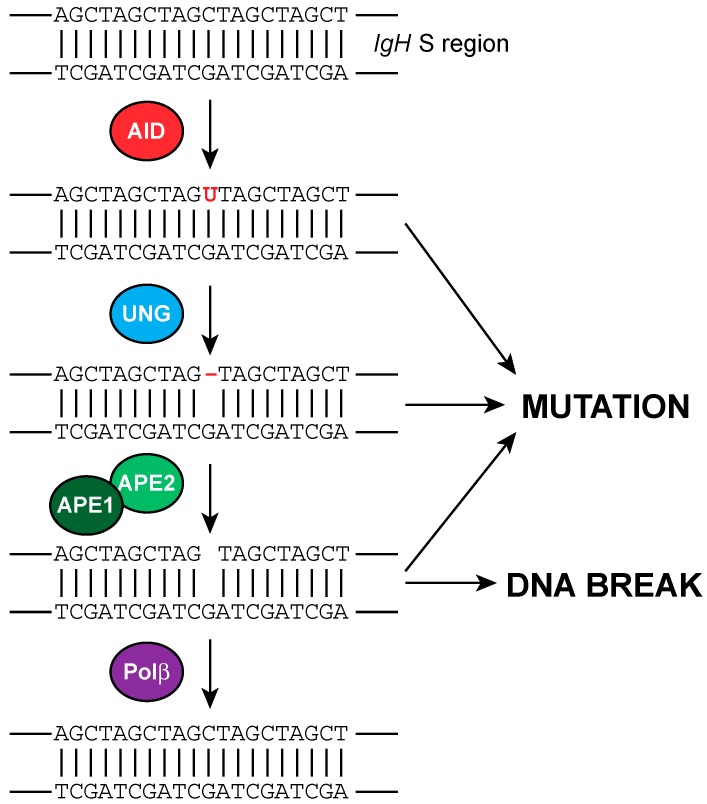
Involvement of BER proteins in CSR. During immunoglobulin (Ig) class switch recombination (CSR) activation-induced cytidine deaminase (AID) converts cytosines into uracils (shown in red font color) in the *Ig* heavy chain (*IgH*) switch (S) regions. Processing of the resulting uracils lead to either DNA mutations or DNA breaks, or can be faithfully corrected. AID-instigated uracils are processed by the base excision repair (BER) pathway. Replication across a uracil can lead to C-to-T/G-to-A transition mutations. Alternatively, the uracil is removed by uracil-DNA-glycosylase (UNG) leading to an apyrimidinic/apurinic (AP) site (shown as red dash), which, when replicated, result in either transition or transversion mutations. The AP site can also be cleaved by AP endonuclease 1 and 2 (APE1/2), which when sufficiently close on either strand results in the formation of a DNA break, or when spaced further apart require the mismatch repair (MMR) pathway to be converted into DNA double-strand breaks. DNA polymerase β (Polβ) is required for faithful repair of the APE1/2 nicked DNA backbone, and would thereby diminish Ig CSR.

Following encounters with antigens, B cells rearrange their constant region genes in the *IgH* locus to switch from expressing one class of Ig, such as IgM, to IgG, IgA, or IgE antibodies that have defined roles in the immune system. This process is called class switch recombination (CSR). CSR occurs by a deletional recombination event between two different switch (S) regions, which are located upstream of each constant region in the *IgH* locus, except for Cδ. CSR can be defined in two phases: the introduction of DSBs in the donor S region and an acceptor S region, followed by ligation between these distal breaks [82]. S regions are GC-rich and have a high density of WGCW (A/T-G-C-A/T) motifs, which are preferred targets of activation-induced cytidine deaminase (AID), an enzyme that is critical for the formation of DSBs at the S region [83]. Formation of γH2AX foci, which are markers of DSBs, have been observed at the *Ig* locus of wild-type (WT) splenic B cells that are stimulated to undergo CSR, but not in AID-deficient B cells [84]. AID deaminates cytosines in the S regions of the *Ig* locus, thereby converting them to uracils. Processing of the resulting U:G mismatches by base excision repair (BER) and mismatch repair (MMR) pathways leads to the formation of DSBs, which can be subsequently repaired by NHEJ [85] (Figure 5). Uracils are actively removed from the DNA by uracil-DNA-glycosylase (UNG) resulting in the formation of apurinic/apyrimidinic (AP) sites. There are four different uracil DNA glycosylases (UNG, SMUG1, MBD4, and TDG), but UNG is the most crucial for CSR, since UNG deficiency reduces CSR to 95–99% in both human and mice [83,86,87,88]. Moreover, UNG is mostly active on single-stranded DNA, which is also the main substrate for AID. In contrast to the well-established activities of UNG, it has been suggested by Begum et al. that a UNG mutant deficient in uracil-removal activity can still promote CSR [89]. However, as has been argued by Stiver et al. this can be due to the low level of residual U-removal activity of the UNG mutant [90]. The residual class switching in *Ung−/−* mice can be dependent on Smug1, since serum IgG3, IgG2b, and IgA are greatly diminished in *Ung−/− Smug1−/−* mice. However, there is still a detectable level of switched isotypes in *Ung−/− Smug1−/−* mice, which suggests a role for other DNA glycosylases [91]. In line with this, a recent report by Grigera et al. showed that the deletion of exon 8 of *Mbd4* reduced the formation of DSBs and impaired CSR [92].

Both UNG and SMUG1 are monofunctional glycosylases that remove the uracil base. However, they are unable to cleave the DNA deoxyribose phosphate backbone [93]. Apurinic/apyrimidinic endonucleases (APEs) are required for the formation of nicks in the DNA backbone. APE1 is an essential protein and is the major AP endonuclease in eukaryotes [94]. If the nicks created by AP endonuclease are in close proximity on both strands, then it results in DSB formation required for CSR [95] (Figure 5). Ape1 is pivotal for early mouse embryogenesis and development [96]. Though *Ape1+/−* mice are viable, they have DNA repair defects [97]. APE1 is the major AP endonuclease in mammals with a weak 3′–5′ exonuclease and 3′phosphodiesterase activity [98]. In vivo data showed that APE1 is also capable of cleaving double-stranded DNA containing a U:G mismatch without the need for UNG or SMUG [99]. However, the large CSR defect in *Ung−/−* mice suggests that this is very unlikely to happen during CSR [86].

APE2 is the second AP endonuclease in mammalians with weaker AP site-specific and 3′-nuclease activities [98]. In contrast to Ape1, Ape2 is not required for mouse embryogenesis, and studies have shown that its function is restricted to lymphoid compartments [100,101]. Both Ape1 and Ape2 are required for CSR. In the study by Guikema et al. it was shown that switching to IgG2b and IgG3 was decreased about 77% in *Ape1+/−* splenic B cells compared to WT. *Ape2Y/−* splenic B cells (*Ape2* is located on the X-chromosome), like *Ape1−/− Ape2Y/−* double-deficient (DBL), showed a reduction to 65% of WT. IgG1, IgG2a, and IgG2b isotypes reached significant reductions in *Ape2Y/−* B cells, whereas all isotypes were significantly reduced in DBL B cells. [95]. Residual CSR in DBL splenic B cells could be due to the remaining *Ape1* allele in these cells, and suggest that both Ape1 and Ape2 are involved in CSR by cutting at AP sites, which will be converted to DSBs required for CSR.

DBL cells also showed a severe reduction in the frequency of S region DSBs, having almost as few as *Aid−/−* cells, underscoring the role of both AP endonucleases in the induction of AID-induced DSBs [95,102]. However, deletion of *Ape2* in the mouse lymphoma cell line CH12-F3, which can be activated to undergo CSR in vitro, gave different results. In CH12-F3 B cells in which deletion of *Ape1* was achieved successfully, CSR was reduced to 20% of WT CH12-F3 cells [103]. However, *Ape2* deficiency had no effect on CSR in CH12-F3 cells [104]. Curiously, Xu et al. did not find a reduction of AID-induced S region DSBs in *Ape1*-deficient CH12-F3 cells [103]. This discrepancy might be explained by the study from Masani et al. in which they discovered that CH12-F3 cells have three copies of the *Ape1* gene [104] and the deletion of *Ape1* or *Ape2* alone had no effect on CSR in this cell line. Therefore, the residual level of Ape1 in these cells could be sufficient for CSR.

After the incision of the DNA backbone by AP endonuclease, the deoxyribose phosphate group (dRP) is still attached to the 5′ end of the break. DNA Polβ is known to have lyase activity, which excises this dRP group to process the single-strand break (SSB) [105]. Normally, DNA Polβ accurately replaces the excised nucleotide, and Ligase III-XRCC1 seals the phosphodiester backbone [93]. Thus, these activities of Polβ predict that it negatively regulates CSR (Figure 5). In agreement with this, Wu and Stavnezer showed that *Polβ−/−* B cells had slightly increased CSR to IgG2a, IgG2b, and IgG3 in vitro, and had more S region DSBs upon CSR activation. The effect of Polβ deficiency was more apparent when CSR was induced sub-optimally by reducing the concentrations of the switch inducers lipopolysaccharide (LPS) and cytokines. These results suggest that AID-instigated lesions required for CSR are counteracted by Polβ, at least in an in vitro setting. It was suggested that, during CSR, Polβ is overwhelmed due to the high load of AID-induced lesions in the S regions, so some nicks remain unrepaired, which provides the source for the formation of DSBs [106]. Whether this holds true in vivo remains to be established.

## 8. Role of Mismatch Repair Factors in Conversion of SSBs to DSBs

Another DNA repair mechanism that was shown to be involved in CSR is the mismatch repair pathway (MMR). It was demonstrated that in vitro CSR is decreased in splenic B cells from mice deficient for the MMR components Msh2, Mlh1, Pms2, and Msh6 [107,108]. MMR was shown to be involved in the formation of AID-instigated S region DSBs, indicating that this pathway, which is normally involved in repairing DNA lesions, is somehow subverted into causing DNA damage during CSR [109]. An important insight into how MMR is involved in DSBs came from crossing Msh2-deficient mice to mice that lacked most of the tandem repeat sequences of the Sμ region (SμTR). In the *Msh2−/− SμTR−/−* mice, CSR was nearly ablated, whereas in the single-deficient mice CSR was reduced 2- to 3-fold [110]. The SμTR region contains most of the closely spaced AID hotspots that, when nicked on both strands, does not require any further processing to generate a DSB. In the *SμTR−/−* mice, the AID hotspots are, however, more distantly spaced. MMR was shown to be required for processing these distant nicks by recruitment of exonuclease 1 (Exo1), which excises a single-strand segment starting from a nick located 5′ from a U:G mismatch that is generated by AID. Exo1 can then excise a segment until it reaches a nick on the opposite strand, resulting in a DSB [110,111,112]. Other MMR proteins such as Msh3 and Msh5, which are involved in the recognition of bulky DNA lesions and crossing-over during meiosis, respectively, do not have a role in CSR [108,113].

## 9. DNA Damage Response Regulates CSR and AID

**Figure 6:**
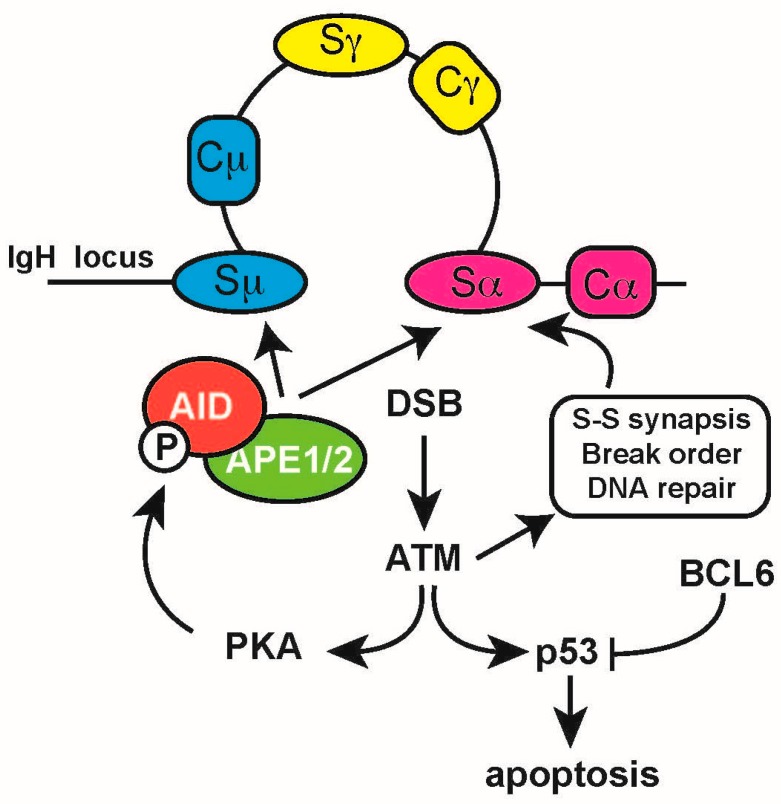
DNA damage triggers an ATM- and phosphorylation-dependent S region DSB feedforward loop. Activation-induced cytidine deaminase (AID) instigates DNA double-strand breaks (DSBs) in the switch (S) regions upstream of each immunoglobulin (Ig) constant (C) region in the heavy chain locus (*IgH*). Activation of ataxia telangiectasia mutated (ATM) kinase by DSBs triggers the protein kinase A (PKA) -dependent phosphorylation of AID on serine 38 (represented by P in the black circle), which enhances the interaction of AID with apurinic/apyrimidinic endonuclease 1 (APE1), which is involved in processing of AID lesions into DSBs. ATM also regulates the synapsis of S regions required for intrachromosomal recombination; the order in which the S region DSBs occur; the recruitment of DNA repair factors necessary for resolvement of the recombination (indicated in the black frame). Simultaneously, ATM may trigger p53-dependent activation of apoptosis, which is counteracted by the B-cell lymphoma 6 (BCL6) protein. Arrows represent positive regulation (activation); T-bar represent negative regulation (inhibition).

The AID-instigated DSBs in the S regions activate and recruit DDR factors, which play important roles in resolving these lesions. During CSR, S region DSBs lead to the instant recruitment of the Mre11-Rad50-Nbs1 (MRN) complex, which in turn recruits the DDR master regulator ATM [114]. ATM phosphorylates histone H2AX and the Nijmegen breakage syndrome 1 (NBS1) proteins at the sites of the S regions DSBs [84], recruiting and phosphorylating several other DDR factors, such as p53 binding protein 1 (53BP1) and mediator of DNA damage checkpoint 1 (MDC1). The importance of these events is illustrated by the CSR defects in B cells deficient for these proteins. CSR in *Atm−/−* and *H2ax−/−* splenic B cells is reduced to about 20–30% of WT upon in vitro activation, and CSR nearly is ablated in *53bp1−/−* splenic B cells [115,116,117,118,119]. Whereas *Mdc1−/−* splenic B cells show a somewhat milder phenotype regarding CSR [119,120]. For efficient CSR, Sμ and the downstream S region targeted for recombination need to be brought in close proximity, forming an S–S region synapse, in part determined by S region germline transcription [121]. Surprisingly, ATM and 53BP1 have been implicated in the complex orchestration of these topological changes within the *IgH* locus, independent of their roles in DNA repair. In the absence of ATM, DSBs accumulate in Sμ, while DSBs in the downstream S region are reduced. It was suggested that AID first introduces DSBs in Sμ, which then triggers S–S synapse formation and subsequent introduction of DSBs in the downstream S region [119]. In addition, 53BP1 was recently shown also to be required for S–S synapse formation and for the regulation of the order in which DSBs occur in the S regions [122,123]. These non-canonical activities of the DDR proteins illustrate that B cells have evolved complex strategies to utilize DNA damage sensing and repair systems for *Ig* diversification (Figure 6).

Another interesting example of how B cells use the DDR for diversification was recently uncovered by the Chaudhuri group, showing that the ATM kinase is a pivotal player in a positive feedback loop that promotes DSBs in S regions during CSR. They demonstrated that DSBs provoke the ATM-dependent activation of protein kinase A (PKA), which was previously implicated in regulating AID by serine 38 phosphorylation [124]. DSB-triggered AID phosphorylation promoted its interaction with APE1, thereby further enhancing S region DSB formation [125]. These results show that activation of the DDR in B cells undergoing CSR stimulates the engagement of BER proteins, promoting AID-dependent DNA damage (Figure 6).

## 10. CSR and Chromosomal Translocations

Physiological DSBs that are intermediates in CSR can be potential substrates for chromosome translocation in mammalian cells [126,127]. Reciprocal chromosomal translocations are the most common type of translocation in lymphoid malignancies. In most of the B-cell lymphomas, these translocations result in dysregulated expression of oncogenes that are juxtaposed to the *Ig* enhancers [74]. An “infamous” example is the *MYC/IGH* translocations found in Burkitt′s lymphomas in which *IGH* regulatory elements are misplaced upstream of the *MYC* proto-oncogene [128]. Other examples of translocation between *IGH* and other oncogenes are *BCL2* in FL, *BCL6* in diffuse large B cell lymphoma (DLBCL) and *CCND1* (Cyclin D1), *CCND3* (Cyclin D3), *FGFR3/MMSET* (Fibroblast growth factor receptor 3/multiple myeloma SET domain), and *MAF* (c-maf) in multiple myeloma (MM). [74,129]. The mechanisms of chromosomal translocations have already been reviewed elsewhere extensively [76]. The majority of B-cell lymphomas originate from mature B cells or postgerminal center B cells, where AID is normally expressed. Unlike RAG that introduces DSBs at specific sites (RSS sequences), AID can deaminate cytosines in nearly any sequence context preferably in the RGYW motif [130,131]. There is compelling evidence indicating the involvement of AID in genomic instability in B cells and development of chromosomal translocation. The report by Ramiro et al. showed that *Aid+/−* mice crossed to an interleukin-6 transgenic (Il6-Tg) background harbored B cells carrying the *IgH/c-myc* translocation, whereas these were undetectable in *Aid−/−* Il6Tg mice, indicating the contribution of AID in genome instability [132]. Deep sequencing analysis on WT and AID-deficient mice by Liu et al. [133] furthermore showed that AID can act broadly throughout the genome in mouse B cells and deaminates numerous tumor-related genes including *Myc, Pim1, Pax5, Ocab, H2afx, Rhoh,* and *Ebf1*. In line with these data, the report by Staszewski et al. identified hundreds of reproducible, AID-dependent DSBs in mouse splenic B cells upon induction of CSR in culture. Interestingly, some of the AID-induced DSBs occur at sites that are known to be translocated or amplified or deleted in human B-cell lymphomas, such as the B-cell lymphoma 11a gene (*Bcl11a/Evi9*) [114]. The development of high-throughput genome-wide translocation sequencing (HTGTS) technique by the Alt laboratory provided insight into the mechanism that controls chromosomal translocation [134]. At the same time, Klein and coworkers developed another technique, translocation capture sequencing (TC-seq), which led to the identification of hotspots for AID-mediated translocations in mature B cells [135]. Along these experimental lines, using mouse B lymphocytes, Hakim et al. suggested that the frequency of DSBs directly accounts for the rate of chromosomal translocation [136]. Normally, the high-fidelity repair pathways (BER and MMR) counteract off-target effects of AID. However, under some conditions, such as environmental factors including cellular stress, hypoxia, and viral infections, and intrinsic factors such as altered expression of error-prone polymerases, the balance between high-fidelity and error-prone DNA repair pathways might be disrupted, which changes the outcome of AID-induced lesions [133]. Moreover, malignant B cells may have lost the balance between the formation and repair of AID-induced lesions due to enhanced AID activity, or reduced high-fidelity repair.

## 11. Antibody Diversification by SHM

It is estimated that the number of different antibodies that can be produced in human is ~10^9^, which exceeds the coding capacity of the inherited genome. V(D)J recombination is known to provide around 10^5^–10^6^ different antibody specificities in both mice and human. However, it is the SHM that provides high affinity antibodies against foreign antigens [130]. Once exposed to invading pathogens in the secondary lymphoid organs, B cells engage in the germinal center reaction and start to express AID. AID increases the affinity of antibodies by introducing point mutations at a frequency of around 10^−3^ per base pair per generation in the variable regions of the rearranged *Ig* genes [14]. The number of mutations and the level of SHM both depend on several factors, including the nature of the antigen, route of exposure, and selection. For example, neutralizing antibodies against influenza virus can accumulate 30–40 mutations at the complementarity determining regions (CDR) of the expressed Ig, whereas antibodies against HIV bear up to >100 mutations, which suggests that these mutations are accumulated over time through multiple rounds of germinal center reactions [137,138]. Moreover, insertions and deletions are introduced during SHM and help increase the affinity of Abs [139,140]. B cells with increased BCR affinity are positively selected and undergo iterative cycles of cell division, whereas those with declined BCR affinity are depleted from the population [141,142].

## 12. Involvement of BER and MMR in SHM

**Figure 7:**
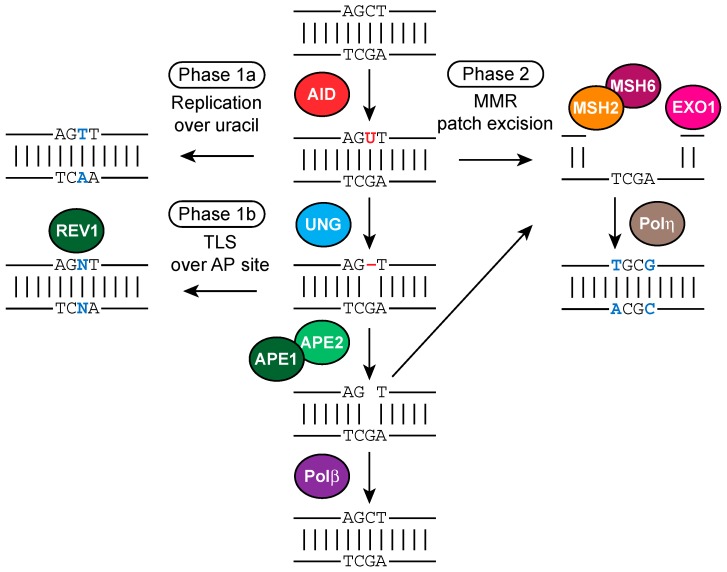
Molecular mechanism of Phases 1 and 2 SHM, involving MMR and BER proteins. Processing of AID-instigated uracils (shown in red font color) determine the somatic hypermutation (SHM) spectrum (mutations are shown in blue font color). Replication over uracils result in C-to-T/G-to-A transition mutations (Phase 1a SHM). Uracil removal by uracil-DNA-glycosylase (UNG) followed by replication over the apurinic/apyrimidinic (AP) site (shown as red dash) by translesion synthesis (TLS) DNA polymerases such as REV1 result in transition or transversion mutations are C/G base pairs (bp) (Phase 1b SHM). Recognition of AID-instigated U:G mismatches by the mismatch repair (MMR) pathway components Mut S homolog 2 (MSH2) and MSH6 and recruitment of DNA exonuclease 1 (EXO1) lead to patch excision, and subsequent recruitment of DNA polymerase η (Polη), which is error-prone particularly at A and T bps, results in A/T mutations (Phase 2 SHM). AP sites can be cleaved by AP endonuclease 1/2 (APE1/2), the resulting nicks may serve as entry points for EXO1 and thereby stimulate Phase 2 mutations. Alternatively, DNA Polβ triggers faithful repair of AID-induced lesions.

Like CSR, SHM is mechanistically initiated by AID and both BER and MMR components are required for this process to occur [130,143]. During SHM, mutations are introduced in two distinct phases, depending on the context of U:G mismatch processing (Figure 7). Mutations at G:C bps are termed Phase 1 mutations, and A:T mutations occur in Phase 2. Phase 1 mutations can be triggered by replication over AID-generated U:G mismatches, giving rise to C-to-T and G-to-A transition mutations (Phase 1a). For these mutations to take place U:G mismatches either need to escape faithful repair in the G1 phase, or AID deaminates cytosines in S phase. Several reports argue for the former possibility as AID is degraded more quickly in S phase than in the G1 phase, and AID-dependent DSBs have been predominantly detected in the G1 phase [109,144]. Phase 1a mutations do not depend on BER or MMR, in contrast to Phase 1b and Phase 2 mutations (Figure 7).

During Phase 1b, the U:G mismatch is processed by the BER component UNG, resulting in the formation of an AP site, which, when replicated by translesion synthesis (TLS) polymerases such as Rev1, results in G:C transversion mutations, as shown by the dramatic shift towards transition mutations in Ung-deficient mice, while having only a moderate impact on A:T mutations. Besides, the overall mutation frequency remained unchanged [86,88,145]. The effect of Ung deficiency on the reduction of G:C transversion mutations highlights the role of Rev1, which was shown to be responsible for the induction of G:C transversion mutations during SHM in mice [146]. APE1 and APE2 are two other BER components that play important roles during SHM. AP site incision by APE1 or APE2 may instigate correct repair of AID-induced lesions by providing a substrate for Polβ, which is a high-fidelity polymerase. However, Ape1 and Polβ are expressed at low levels in germinal center B cells, thereby allowing error-prone repair of AID lesions [147,148].

A:T mutations are generated during Phase 2 and require MMR (Figure 7). The U:G mismatches can be recognized by MSH2/MSH6 heterodimers, which results in recruitment of MutLα, composed of MLH1 and PMS2 that can nick the DNA on 5′ site of the mismatch via PMS2 endonuclease activity [149]. Subsequently, the PCNA-associated EXO1 exonuclease removes the mismatch by a 5′ to 3′ exonuclease activity. Recruitment of low fidelity polymerases (Polη, ζ, ι) through PCNA ubiquitination instead of high fidelity DNA polymerases initiates mutagenic repair that mostly affect A:T bps. [150,151,152,153]. Strikingly, MMR is typically a high-fidelity repair pathway, but is subverted to an error-prone non-canonical type of MMR (ncMMR) in germinal center B cells. The error-prone nature of ncMMR seems to be associated with the G1 phase in which it is executed, and relies on lysine 164 monoubiquitination of PCNA [154,155]. Deficiency in Msh2 and Msh6 in mice has been shown to result in decreased mutations at A:T bps (reduced to 5%–25% of WT) [145,156,157,158,159]. It was suggested that the residual A:T mutations observed in Msh2-single-deficient mice are generated by Ung and recruitment of polη, since combined Ung/Msh2, Ung/Msh6, or Msh2/polη deficiency resulted in the total ablation of A:T mutations [160,161]. These finding support the hypothesis that there is a collaboration between BER and MMR rather than competition. Exo1 is the first exonuclease known to participate in SHM and physically interacts with Mlh1. A marked decline at A:T mutations has been observed in mice deficient in Exo1 [162]. Exo1 requires a nick as an entry point for its exonuclease activity. The identity of the nucleases responsible for providing the nick during SHM has not been firmly established. Possible candidates may be Pms2 and/or Ape2.

Interestingly, the BER component Ape2 was implicated in Phase 2 mutations. In contrast to Ape1, Ape2 is expressed in germinal center B cells, and Stavnezer et al. showed that the total mutation frequency is reduced about 2-fold in *Ape2Y/−* mice. Most strikingly, the proportion of mutations at A:T bps is reduced in *Ape2Y/−* mice, comparable to level in *Ung−/−* mice. Moreover, deficiency of both Ung and Ape2 leads to a more dramatic decrease in mutation at A:T, which indicates that ssDNA created by Ape2/Ung can be used as an entry point for ncMMR and TLS polymerases to induce mutation at A:T bps [147] (Figure 7).

## 13. Aberrant SHM Activity and its Contribution to Genomic Instability

AID-induced mutations are predominantly restricted to the *Ig* genes. However, AID can also target a subset of transcriptionally active genes outside of the *Ig* loci in human and mouse B cells, including proto-oncogenes such as *Bcl6* and *Myc* [163,164]. Mutation analysis in several AID target genes in germinal center B cells from *Ung−/− Msh2−/−* mice showed that a wide variety of genes, scattered throughout the genome, harbor AID-dependent mutations. These results show that the genome is protected from widespread AID-induced mutations by the combined activities of the BER and MMR pathways [164]. Additional evidence that AID-induced mutations are involved in lymphomagenesis has been provided by a study showing that AID deficiency prevented Bcl6-dependent germinal center B-cell derived lymphomas in a mouse model, whereas it had no impact on lymphomas derived from pre-germinal center B cells in a Myc-driven model [165].

## 14. DDR Regulation in the Germinal Center

**Figure 8:**
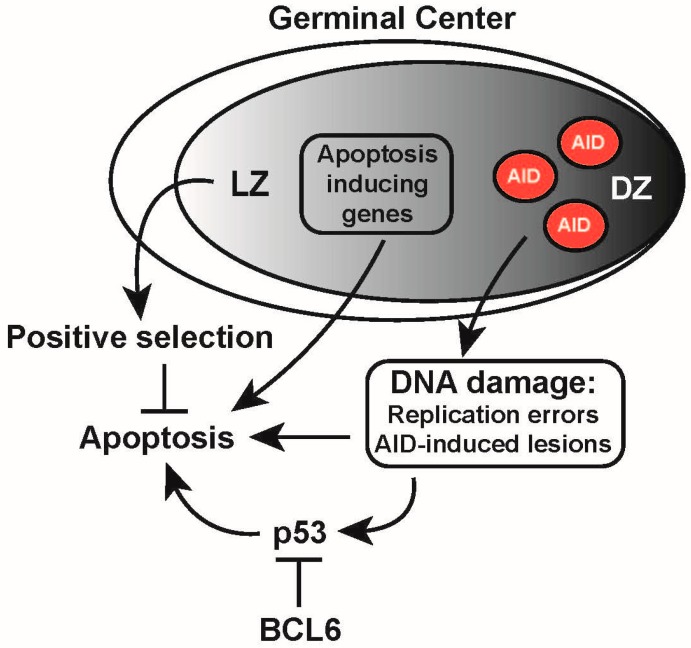
DNA damage response regulation in the germinal center. In the germinal center, centroblast undergo rapid proliferation, and expression of activation-induced cytidine deaminase (AID) is initiated. Moreover, centroblasts in the dark zone (DZ) and centrocytes in the light zone (LZ) are prone to undergo apoptosis as they express apoptosis-inducing genes (indicated in the black frame), such as *FAS* and *BAX*, and lack expression of the anti-apoptotic gene *BCL2*. During affinity maturation in the germinal center reaction, B cells that acquire sufficient T-cell help, based on the affinity of their B-cell receptors, are positively selected and rescued from apoptosis. At the same time, DNA damage caused by replication errors and AID-induced lesions (indicated in the black box) trigger a DNA damage response that may activate a p53-dependent apoptosis response, which is counteracted by the B-cell lymphoma 6 (BCL6) protein. Arrows represent positive regulation (activation); T-bars represent negative regulation (inhibition).

Germinal center B cells are characterized by the expression of Bcl6, which is the master regulator of the germinal center reaction [166]. In addition to regulating/repressing several key transcription factors important in (post-) germinal center B-cell development [167], it also controls the DDR activated by AID-instigated lesions, in a manner similar to that of pre-B cells sustaining RAG-dependent DNA damage [73]. As in pre-B cells, BCL6 has been shown to repress p53 and p21 expression, as well as ATR and CHK1 [168,169,170,171]. This illustrates once again that B cells have evolved mechanisms to evade the DDR to enable *Ig* diversifications (Figure 8).

## 15. AID-Induced Localized Hypermutations and AID Activity in Other Cell Types

Next to disturbances in the delicate balance that B cells need to strike to allow AID-induced DNA damage and the simultaneous repair of such lesions in the *Ig* loci, it is apparent that the role of AID in tumorigenesis is related to the promiscuous targeting properties of this enzyme. The molecular basis of why and how non-*Ig* loci are targeted by AID are only beginning to be understood. Moreover, it has become clear that AID expression and activity is not restricted to B cells and B-cell malignancies, as other cell types and cancer types bear the signs of (aberrant) AID activity. For instance, sequencing of cancer genomes has revealed the presence of localized hypermutations, called kataegis (from the Greek for “thunderstorm”). AID was shown to be responsible for clustered mutations (kataegis) found in chronic lymphocytic leukemias (CLL) and other type of cancers [172,173,174]. These clustered mutations are often associated with sites of DNA rearrangements (like localized chromothripsis) that frequently occur during carcinogenesis [175,176,177]. Based on the type of mutations (>70% C:G–T:A transitions), as well as the sequence context of the mutations, it has been hypothesized that AID is involved in this process by processive cytidine deamination of ssDNA [178]. Human lymphomas derived from germinal center B cells show WRCY hypermutation hotspots, which further indicates the involvement of AID in this process [172]. However, kataegis is distinct from SHM. In contrast to SHM, kataegis occur largely on the same DNA strand and is observed outside of the *Ig* loci, near promoters of off-target genes (>80% in DLBCL) [179]. Since the majority of the mutation in kataegis are C:G–T:A transitions, it suggests that most of these mutations occur during the S phase of the cell cycle by replication over AID-induced uracils [180]. It has been proposed that kataegis can be triggered by processing DSBs by resection during homologous recombination, thereby exposing large stretches of ssDNA, which is the preferred substrate of AID [174,180].

Aberrant AID activity is not only involved in B-cell malignancies but also in various human cancers [181,182,183]. Phenotypic analyses revealed that AID-transgenic mice develop neoplasia in other epithelial tissues, including lung cancer, gastric cancer, and hepatocellular carcinoma (HCC). More interestingly, the *MYC* and *KRAS* genes were the preferential targets of AID in lung and stomach cancers [184]. Subsequent studies by the same group provide a link between the expression of pro-inflammatory cytokines and abnormal expression of AID in colonic epithelial cells. [185,186]. In agreement, dysregulation of AID is associated with accumulation of mutation in the TP53 tumor suppressor gene in gastric cancer [187]. Therefore, regulation and repair of AID-induced lesion is not only important in the *Ig* loci but also outside of the *Ig* loci in non-B cells.

## 16. Concluding Remarks and Future Perspectives

In recent years, much progress has been made in our understanding of the regulation of the *Ig* gene altering events that shape the B-cell repertoire. It has become clear that B cells both engage as well as counteract the DDR and the different DNA repair pathways in order to maximize antigen receptor diversity while minimizing collateral damage. The evolutionary trade-off between adaptive immunity and genomic integrity is underscored by the utilization of DNA repair pathways for mutagenic purposes in B cells. However, despite recent insight, the mechanistic basis for this is not yet entirely clear. Importantly, these phenomena seem not to be entirely restricted to B cells and might contribute to cancer in a more general context [154].

Feedforward and feedback regulatory mechanisms activated by V(D)J recombination and CSR have been uncovered, which predominantly depend on the central DDR sensor kinase ATM, regulating downstream effectors such as NF-κB and PKA [70,71,125]. However, the elucidation of the exact molecular intricacies of these regulatory pathways requires more work. Additionally, whether similar regulatory pathways are at play during SHM, and whether these are localized events that only act at the *Ig* loci or have more widespread implications on the genomic integrity in B cells (and perhaps other cell types), remain important outstanding questions. The circumvention of faithful DNA repair during CSR and SHM in germinal center B cells, and how this is regulated at the *Ig* loci versus the non-*Ig* loci is another interesting and important issue that needs to be studied further.

The conservation of the mechanisms involved in both the utilization and evasion of DDR and DNA repair pathways during antigen receptor diversifications in B cells clearly suggest that it provides evolutionary advantages at the organismal level; however, the exact contribution of each of these pathways and mechanisms to the immune fitness of an animal has not received much attention. Lastly, the identification of additional factors that shape the antigen receptor repertoire by regulating V(D)J recombination, CSR and SHM will offer new ways to improve vaccination strategies and the development of antibody-based therapeutics, and will shed light on the contribution of these processes to genomic instability and oncogenesis.
